# Antifungal Activity of Perillaldehyde on *Fusarium solani* and Its Control Effect on Postharvest Decay of Sweet Potatoes

**DOI:** 10.3390/jof9020257

**Published:** 2023-02-15

**Authors:** Chao Pan, Kunlong Yang, Famous Erhunmwunsee, Bo Wang, Dongjing Yang, Guoquan Lu, Man Liu, Yongxin Li, Jun Tian

**Affiliations:** 1 School of Life Science, Jiangsu Normal University, Xuzhou 221116, China; 2 Xuzhou Institute of Agricultural Sciences in Jiangsu Xuhuai District, Xuzhou 221131, China; 3 School of Agriculture and Food Science, Zhejiang A & F University, Hangzhou 311300, China

**Keywords:** root rot, storage, spore viability, mitochondria, reactive oxygen species

## Abstract

Root rot caused by *Fusarium solani* is one of the major postharvest diseases limiting sweet potato production. Here, antifungal activity and the action mode of perillaldehyde (PAE) against *F. solani* were investigated. A PAE concentration of 0.15 mL/L in air (mL/L air) markedly inhibited the mycelial growth, spore reproduction and spore viability of *F. solani*. A PAE vapor of 0.25 mL/L in air could control the *F. solani* development in sweet potatoes during storage for 9 days at 28 °C. Moreover, the results of a flow cytometer demonstrated that PAE drove an increase in cell membrane permeability, reduction of mitochondrial membrane potential (MMP) and accumulation of reactive oxygen species (ROS) in *F. solani* spores. Subsequently, a fluorescence microscopy assay demonstrated that PAE caused serious damage to the cell nuclei in *F. solani* by inducing chromatin condensation. Further, the spread plate method showed that the spore survival rate was negatively correlated with the level of ROS and nuclear damage, of which the results indicated that PAE-driven ROS accumulation plays a critical role in contributing to cell death in *F. solani*. In all, the results revealed a specific antifungal mechanism of PAE against *F. solani*, and suggest that PAE could be a useful fumigant for controlling the postharvest diseases of sweet potatoes.

## 1. Introduction

The Food and Agriculture Organization of the United Nations reported that the yield of sweet potato (*Ipomoea batatas* Lam.) was 88.87 million tons all over the world in 2021, and about three quarters of this yield came from China [1]. Sweet potato is an important food crop around the world, especially in developing countries, because it can produce more dry matter, protein and minerals per unit area in comparison to cereals [2,3]. Due to containing a number of nutritional factors, such as carbohydrates, carotenes, vitamins, potassium et al., sweet potato roots have been developed into many foods and beverages [4]. However, because they have a high moisture and carbohydrate content, the roots are easily infected by *Fusarium solani*, resulting in postharvest deterioration [5,6].

The filamentous fungus *F. solani* is a plant and human pathogen belonging to the *Fusarium solani* species complex (FSSC), and its sexual state is *Nectria haematococca* [7]. *F. solani* is ubiquitously distributed in soil and decaying plant materials, where it acts as a decomposer [8]. However, *F. solani* is an important pathogen of a number of agriculturally important crops; for instance, soybean, potato and tomato, et al. [9]. *Fusarium* root rot caused by this fungus is one of the major postharvest diseases of sweet potato, particularly in the southeastern United States [5], and the main sweet potato-growing areas in China [3] and the Republic of Korea [10]. Muggy conditions, such as a high temperature (13 to 35 °C) and relative humidity (over 80%), contribute to this disease incidence [5]. It was reported that, in China in 2014 and South Korea in 2017, the incidence of Fusarium root rot on sweet potatoes caused by *F. solani* was 10–20% [10]. Further, *F. solani* is able to cause stem lesions and end rot of sweet potatoes in the process of cultivation, leading to a substantial reduction of sweet potato production [5]. On the other hand, *F. solani* was found to be an opportunistic pathogen causing human diseases, such as fungal keratitis or invasive mycoses [9]. Therefore, controlling the contamination of *F. solani* is a crucial approach to ensure food safety and human health.

Wounding is a prerequisite for an infection of sweet potatoes by *F. solani*. However, sweet potato roots are easily wounded due to a relatively thin and delicate skin during its harvest or transportation to market [6]. So far, some management strategies have been developed to reduce the microbial spoilage of sweet potato roots. Specifically, wound healing in large storage containers soon after harvest has served as an important strategy to minimize *Fusarium* root rot [5]. Nevertheless, due to a lack of large storage containers to implement the wound healing process, farmers prefer to use chemical fungicides to combat the sweet potato spoilage; for example, carbendazim, a cheap broad-spectrum fungicide which is widely used, especially in China [11]. However, the use of carbendazim for the storage of edible roots posed a potential risk to human health [12]. Hence, there is an urgent need to develop green preservatives for the storage of the edible part of plants [13].

Perillaldehyde (PAE), a natural monocyclic terpenoid, is a kind of essential oil (EOs) that is abundant in the perennial herb *Perilla* [14]. PAE has been used as a flavoring agent for foods such as baked goods, meat products and beverages et al. [15]. The foods have been certified as safe by the Food and Agriculture Organization of the United Nations (FAO) and United States Food and Drug Administration (FDA) [16]. Interestingly, PAE presents an effective antifungal activity against some pathogenic and food spoilage fungi, such as *Candida albicans* [17], *Ceratocystis fimbriata* [18] and *Aspergillus flavus* [19]. Thus, PAE holds promise as a novel antifungal agent used in food preservation [16]. However, the antifungal activity of PAE against *F. solani* remains unclear.

It is reported that apoptosis plays a key role in the fungistatic pathway executed by the activity of EOs [16], and the reactive oxygen species (ROS) produced by the mitochondria is a major key marker of the apoptotic process [20]. Our previous work revealed that PAE induced cell apoptosis of *A. flavus* via ROS accumulation [21]. According to our previous results of transcriptome sequencing, PAE drove an inhibition of glycometabolism resulting in the indirect suppression of glutathione synthesis in *A. flavus*, which contributed to reducing the ROS-scavenging capacity, ultimately leading to ROS accumulation [22]. *F. solani* was reported to be efficient in utilizing carbohydrates, by including a large number of multiple-copy coding genes for carbohydrate-active enzymes [7]. The results seemed to imply that *F. solani* may possess a high resistance to PAE due to its high carbohydrate-using capacity. In addition, in clinical practice, the infection of *F. solani* is difficult to treat because *Fusarium* spp. are highly resistant to most antifungals [23], such as amphotericin B and imidazoles [24]. However, whether PAE exhibits effective antifungal activity against *F. solani* in sweet potatoes remains unclear.

In the present study, the antifungal activity of PAE against *F. solani* and its preservative effect on sweet potatoes were estimated. First, the effects of PAE on mycelial growth, spore production and viability were examined. Second, the preservative effect of PAE on sweet potato roots was evaluated. Moreover, the mode of antifungal action of PAE was investigated by detecting the cell membrane integrity, mitochondrial membrane potential (MMP), ROS level and nuclear morphometry. With this information, the essential oil PAE can be recommended as a novel green preservative to limit the amount of postharvest loss of sweet potatoes due to *Fusarium* root rot.

## 2. Materials and Methods

### 2.1. Chemicals, Strain and Plant Materials

The PAE (CAS no. 18031-40-8, purity > 90.0%) was purchased from Tokyo Chemical Industry Co., Ltd. (Tokyo, Japan). The PAE was prepared as 10 × stock solutions in 0.1 % (*v*/*v*) Tween 80 with an ultrasonic wave treatment for 30 min. *F. solani* X14011 was originally separated from the rot spot on sweet potato roots, and obtained from Hebei Academy of Agriculture and Forestry Sciences in China [25]. The fungus was cultured on potato dextrose agar (PDA; 20% potato, 2% dextrose, 1.5% agar) for 7 d at 28 °C. Sweet potato roots of a commercial cultivar Xushu 32 grown for about 150 d were obtained from the experimental station of the Xuzhou Institute of Agricultural Sciences in Jiangsu Xuhuai District in 2022. After harvest, these storage roots were placed into a storage facility, where they were cured at 29 °C for 7 days [5]. After wound healing, the roots were stored at 13 to 15 °C until inoculation.

### 2.2. Determination of Antifungal Activity

The effect of PAE on the mycelial growth of *F. solani* was tested using direct contact and vapor phase contact method [26]. In the direct contact method, 8-mm-diameter mycelial plugs were placed on the center of each PDA plate (9 cm diameter) supplemented with 0.01% Tween 80 and different PAE concentrations of 0.125, 0.25, 0.5, 0.75, 1, 1.25 and 1.5 mL/L. In the vapor phase contact method, mycelial plugs were inoculated on PDA plates containing 15 mL PDA and 80 mL air in the space of these dishes. The PAE was dissolved into methyl alcohol to gain different concentrations of stock solutions [27], and then aliquots of 100 μL stock solutions were pipetted on the inside of the lids of every plate to obtain various PAE concentrations of 0, 0.0125, 0.025, 0.05, 0.075, 0.1, 0.125, 0.15 and 0.175 mL/L in air (mL/L air) in the air space of plates. After that, all plates were sealed with a polythene preservative film, incubated for 9 d at 28 °C and colony diameters were measured every 24 h. To calculate the colony diameters, the following formula was used:

a = b − c(1)

where a: colony diameter (cm); b: measured diameter of colony (cm); c: mycelial plug diameter (cm).

Further, the effect of PAE on the spore productivity of *F. solani* was tested [28]. After the colony diameters were measured, a certain volume of phosphate buffer solution (PBS, pH 7.0–7.2) was added to the plates and then both mycelia and spores were scraped off using a spreading rod. Following, the cells were transferred into 50 mL centrifuge tubes, and shaken violently with a vortex mixer. The spore number of each plate was counted using a hemocytometer under a light microscope.

The effect of PAE on spore viability was tested using a coating method via the contact method and vapor phase method [20,26]. In the contact approach, 100 µL spore suspension (2 × 10^3^ spores/mL) was spread on PDA containing various PAE concentrations of 0, 0.25, 0.5, 1.0 and 1.5 mL/L. In the vapor phase assay, spore suspension was firstly spread on PDA plates, and then the plates were supplemented with 100 mL of PAE stock solutions dissolved in methanol to obtain various PAE concentrations of 0, 0.025, 0.05 0.10 and 0.15 mL/L air. After 5 d of inoculation, the number of colony forming units (CFUs) was counted. To calculate the percentage of conidial survival rate, the following formula was used:

a = b/c × 100%(2)

where a: conidial survival rate (%); b: CFUs of PAE-treated group; c: CFUs of control group.

### 2.3. Determination of the Effect of PAE on Sweet Potato Preservation

**Figure 1:**
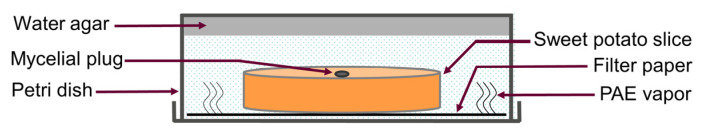
Schema for depicting the assay method determining the effect of PAE on sweet potato preservation.

The preventive effect of PAE on sweet potatoes [29,30] was detected using a vapor phase contact method [31], as shown in Figure 1. Firstly, 15 mL water agar (1.5% agar) was poured into 2 cm high petri dishes. After solidification, the dishes were placed inversely, and one sterile filter paper was placed on the inside of the lids of every plate. Afterwards, healthy sweet potato roots approximately four centimeters in diameter were washed, peeled and sterilized with 1% NaOCl for 10 min. Following this, 1 cm thick slices were cut from the equatorial region of the roots, and these slices were individually placed on the filter paper in each petri dish. Subsequently, 8 mm diameter mycelial plugs were placed on the center of each sweet potato slice. Finally, 100 mL of PAE stock solutions dissolved in methanol was pipetted on the filter paper resulting in a series of PAE concentrations of 0, 0.05, 0.1, 0.15, 0.2 and 0.25 mL/L air in the air in petri dishes. The dishes were incubated for 12 d at 28 °C, and lesion diameters were measured every 3 d. There were four replicates for each treatment. To calculate the percentage of the conidial survival rate, the following formula was used:

a = b − c(3)

where a: lesion diameter (cm); b: measured lesion diameter (cm); c: mycelial plug diameter (cm).

### 2.4. Measurement of Cell Membrane Integrity

Cell membrane integrity was monitored by propidium iodide (PI; Solarbio, Beijing, China) [21]. A spore suspension of 5 × 10^6^ spores/mL was incubated with 1.5 mL/L PAE in a rotary shaker for 2, 4 and 8 h at 28 °C. The spores without PAE treatment were regarded as a control group. After incubation, the spores were washed twice using PBS, and then stained with 1 mL of 10 mg/L PI for 30 min at 28 °C. Finally, the spores were washed three times with PBS, and analyzed using an Accuri^TM^ C6 flow cytometer (BD Biosciences, San Jose, CA, USA).

### 2.5. Determination of MMP

MMP was detected by the fluorescent dye Rhodamine 123 (Rh123; Solarbio, Beijing, China) [18]. A spore suspension (5 × 10^6^ spores/mL) was incubated with 1.5 mL/L PAE, for 0, 2, 4 and 8 h at 28 °C. After incubation, the spores were washed twice, and stained with 100 μg/L Rh123 for 30 min at 28 °C. The spores that were stained with distilled water instead of Rh123 were served as a dye-blank control. Finally, the spores were washed and analyzed by the flow cytometer.

### 2.6. Determination of ROS Level

The ROS production in *F. solani* spores was detected by fluorescent dye 2,7-Dichlorodihydrofluorescein diacetate (DCFH-DA; Sigma-Aldrich, St. Louis, MO, USA) [13]. Spore suspension of 5 × 10^6^ spores/mL was incubated with 1.5 mL/L PAE for 4 and 8 h, 1.5 mL/L PAE plus 80 mM cysteine (Cys) for 4 and 8 h and 80 mM H_2_O_2_ for 8 h at 28 °C. Spores without PAE treatment were regarded as a control group. After treatment, the spores were washed and stained with 10 μM DCFH-DA for 30 min at 28 °C. Finally, the spores were analyzed by the flow cytometer. Meanwhile, spore suspension was serially diluted and spread on PDA. After an incubation for 5 d at 28 °C, the number of CFUs in each group was counted, and the conidial survival rate was calculated using Formula (2).

### 2.7. Determination of Nuclear Morphology

The effect of PAE on the nuclear morphology of *F. solani* was detected using 4′,6-diamidino-2-phenylindole (DAPI; Solarbio, Beijing, China) [18]. A spore suspension (5 × 10^6^ spores/mL) was incubated with 0.75 and 0.15 mL/L PAE, 0.75 or 0.15 mL/L PAE plus 80 mM Cys and with 80 mM H_2_O_2_ for 12 h at 28 °C. After incubation, the spores were stained with 10 mg/L of DAPI for 30 min at 28 °C. After they were washed, these spores were placed on a glass slide and examined using a fluorescence microscope (Leica, Wetzlar, Germany). In addition, the spore suspension was diluted, and spread on PDA plates. After an incubation, the number of CFUs was counted, and the conidial survival rate was also calculated.

### 2.8. Statistical Analysis

The pathogenicity assay was carried out in quadruplicate, and the other assays were performed in triplicate. The results are expressed as mean ± standard deviations (SD), and the statistical significance was calculated by a one-way ANOVA with Duncan multiple range tests using SPSS 21 software (IBM, Chicago, IL, USA). Different letters indicated statistically significant differences at *p* < 0.05.

## 3. Results

### 3.1. Antifungal Activity of PAE against F. solani

**Figure 2:**
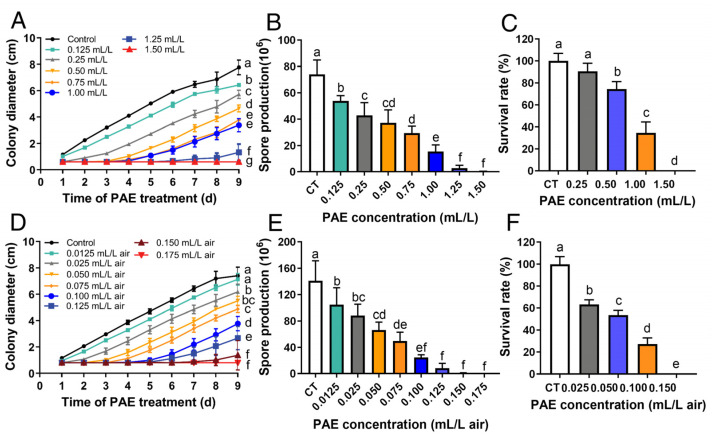
Effects of PAE on mycelial growth, spore production and viability of *F. solani*. Colony diameter (**A**) and spore production (**B**) analyzed by contact method after mycelial plugs were incubated with different concentrations of PAE for 9 d. Colony diameter (**D**) and spore production (**E**) evaluated via vapor phase method after mycelial plugs were incubated with indicated concentrations of PAE for 9 d. Spore survival rate assessed by contact method (**C**) and vapor phase method (**F**) after spores were incubated with different concentrations of PAE for 5 d. All data are presented as mean ± standard deviations (*n* = 3). Different letters indicate statistical significant differences (*p* < 0.05) calculated by one-way ANOVA.

The antifungal activity of PAE against *F. solani* was evaluated by detecting mycelial growth, spore production and viability. Diameters and spore productivity of the colonies that were treated with different concentrations of PAE for 9 d were measured via the contact method and vapor phase contact method. In the contact method, 0.125 mL/L PAE showed an inhibitory effect (*p* < 0.05) on mycelial growth and spore production (Figure 2A,B). The inhibitory effects of PAE on mycelial growth and spore production were in a dose-dependent manner. Notably, mycelial growth was completely inhibited as the PAE concentration reached 1.5 mL/L, and the spore production was also inhibited. This result indicated that the minimal inhibitory concentration (MIC) of the contact method against *F. solani* was 1.5 mL/L PAE. In the vapor phase method, mycelial growth and spore production were suppressed by PAE in a dose-dependent manner (Figure 2D,E). It is worth noting that 0.15 mL/L air PAE inhibited (*p* < 0.05) mycelial growth for 7 d, and 0.175 mL/L air PAE completely inhibited the mycelial growth of *F. solani* (Figure 2D), indicating that the MIC of the vapor phase method against *F. solani* was 1.5 mL/L PAE. Meanwhile, the spore production was also significantly inhibited (*p* < 0.05) under the PAE concentrations of 0.15 and 0.175 mL/L air (Figure 2E). Both assays above proved that PAE, especially PAE vapor, possesses effective inhibitory activity against the mycelial growth and spore production of *F. solani*.

Spore viability was tested using a coating method via the contact method and vapor phase contact method after the spores were treated with different concentrations of PAE for 5 d. In the contact method, the PAE concentration reached 0.5 mL/L which showed an obvious inhibitory effect (*p* < 0.05) on spore survival rate (Figure 2C). The inhibition degree increased as the PAE concentration increased. Exposure to 1.5 mL/L PAE completely inhibited the spore viability. In the vapor phase method, the PAE concentration reached 0.025 mL/L air, which showed an inhibitory effect (*p* < 0.05) on spore survival rate, and 0.15 mL/L PAE completely inhibited the spore viability (Figure 2F). Hence, PAE has a noteworthy inhibitory ability against spore viability in *F. solani*.

### 3.2. Effect of PAE on Sweet Potato Preservation

**Figure 3:**
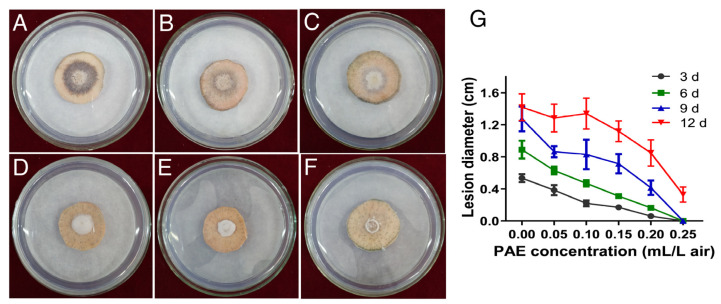
Effect of PAE on sweet potato spoilage caused by *F. solani*. The sweet potato slices inoculated with mycelial plugs were exposed to different PAE concentrations of 0 (**A**), 0.05 (**B**), 0.10 (**C**), 0.15 (**D**), 0.20 (**E**) and 0.25 (**F**) mL/L air for 12 d at 28 °C. Line chart of lesion diameter on these slices (**G**). Error bars represent the mean standard deviation of four replicates.

To evaluate of anti-decay effect of PAE vapor treatment, a pathogenicity assay of *F. solani* on sweet potatoes was carried out. The sweet potato slices inoculated with mycelial plugs were exposed to various concentrations of PAE for different days. The sweet potato slices without the PAE treatment decayed seriously, and a dark and sunken lesion appeared on their surface (Figure 3A). The result proved that the strain *F. solani* X14011 has a strong pathogenicity on sweet potato roots. A PAE concentration of 0.05 mL/L air exhibited a slight inhibitory effect on the expansion of the decayed diameter (Figure 3B). The inhibition degree increased as the PAE concentration increased. When the PAE concentration reached 0.25 mL/L air, sweet potato spoilage could be suppressed up to 9 d (Figure 3F,G). Therefore, PAE showed a notable preservative effect on sweet potato roots infected by *F. solani*.

### 3.3. Effect of PAE on Cell Membrane Integrity

**Figure 4:**
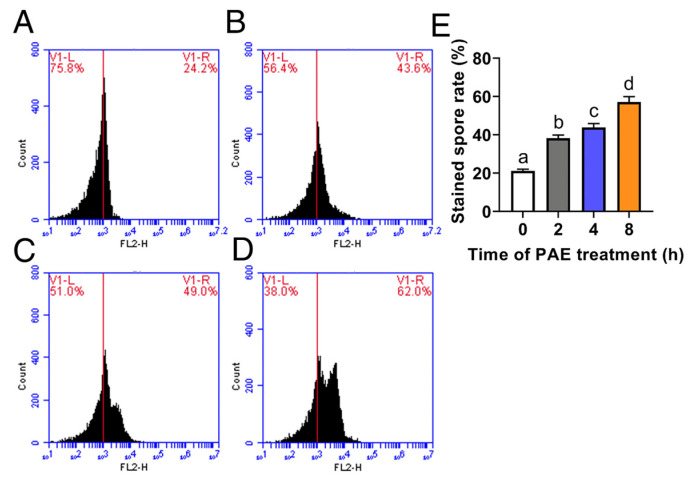
Distribution of fluorescence intensity values of cell membrane integrity in *F. solani* detected by PI staining, after spores exposed to 1.5 mL/L PAE for 0 (**A**), 2 (**B**), 4 (**C**) and 8 (**D**) h. Bar graph of rate of stained spores (**E**). Values are mean ± standard deviations (*n* = 3). Different letters indicate statistically significant differences (*p* < 0.05) calculated by one-way ANOVA.

Cell membrane integrity was estimated using PI staining by a flow cytometer after *F. solani* spores were exposed to 1.5 mL/L PAE for different hours. In the control group without PAE treatment, the spores were not stained with PI, and their fluorescence intensity values were mainly about 10^3^ (Figure 4A). After exposure to PAE for 2 h, the rate of stained spores rose from 21.1% in the control group (Figure 4A) to 38.2% (Figure 4B) with an 81.0% increase (*p* < 0.05). The rate of stained spores increased as the PAE exposure time extended (Figure 4E). As the treatment time of PAE reached 8 h, an obvious intensity peak appeared at the value of 10^4^ on the horizontal axis, and the stained spore rate rose to 57.1% (*p* < 0.05, Figure 4E). This assay demonstrated that PAE caused obvious damage to cell membrane integrity in *F. solani*.

### 3.4. Effect of PAE on MMP

**Figure 5:**
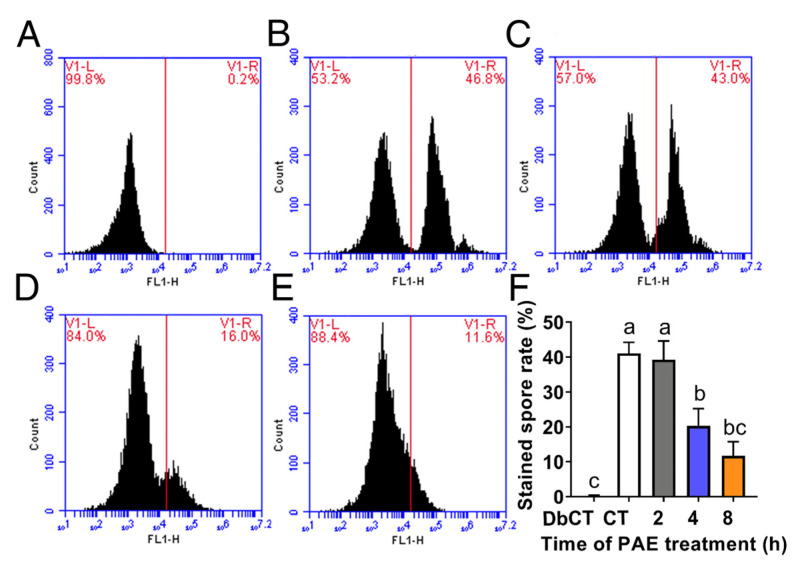
Distribution of fluorescence intensity values of mitochondrial membrane potential (MMP) in *F. solani* detected by Rh123 staining. Spores were exposed to 1.5 mL/L PAE for 0 as control (CT) (**B**), 2 (**C**), 4 (**D**) and 8 (**E**) h. The spores that were stained with water instead of Rh123 were served as a dye-blank control (DbCT) (**A**). Bar graph of stained spore rate (**F**). Values are mean ± standard deviations (*n* = 3). Different letters indicate statistically significant differences (*p* < 0.05) calculated by one-way ANOVA.

MMP was detected using Rh123 staining by a flow cytometer after *F. solani* spores were exposed to 1.5 mL/L PAE for different hours. In the control group, there were two fluorescence intensity peaks (Figure 5B), of which the abscissa value of the first peak was approximately 10^3^, similar to the value of the only fluorescence intensity peak in the dye-blank control (Figure 5A), and of which the abscissa value of another intensity peak that was produced by the stained spores was about 10^5^, indicating that these spores had a high value of MMP. After treatment with PAE for 4 h, the peak area obviously decreased (*p* < 0.05), suggesting that PAE caused a reduction of MMP in *F. solani* spores. As the PAE treatment time reached 8 h, the second fluorescence intensity peak of the Rh123-stained spores almost disappeared, and the abscissa value of the first peak was similar to the fluorescence intensity peak in the dye-blank control (Figure 5A,E), implying that its MMP almost disappeared. So, the above result demonstrated that PAE destroyed MMP in *F. solani*.

### 3.5. Effect of PAE on ROS Accumulation

**Figure 6:**
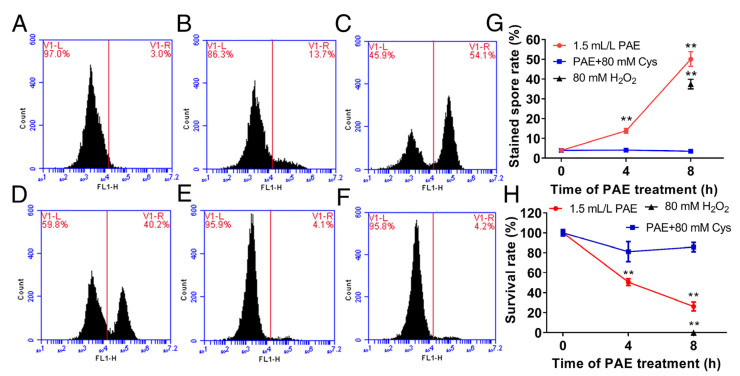
Effect of PAE on ROS level in *F. solani* detected by DCFH-DA staining. Spores were exposed to 1.5 mL/L PAE for 4 (**B**) and 8 (**C**) h, 80 mM H_2_O_2_ for 8 (**D**) h, and to 1.5 mL/L PAE plus 80 mM cysteine (Cys) for 4 (**E**) and 8 (**F**) h. Spores without PAE treatment were served as control (**A**). Line charts of rate of stained spores (**G**) and survival rate of spores in every experimental group (**H**). Values are mean ± standard deviations (*n* = 3). ** indicates *p* < 0.01 compared with the control group calculated by one-way ANOVA.

The ROS level was measured using DCFH-DA staining by a flow cytometer after the spores of *F. solani* were exposed to 1.5 mL/L PAE for different hours. In the control group without PAE treatment, there was only one fluorescence intensity peak (Figure 6A). After incubation of PAE for 4 h, the rate of stained spores significantly (*p* < 0.05) increased (Figure 6B). The rate of stained spores increased as the time of PAE exposure extended (Figure 6G). After incubation of PAE for 8 h, there was a new-appeared fluorescence intensity peak that was located near 10^5^ on the horizontal axis (Figure 6C), of which the abscissa value was similar to the abscissa value of the second fluorescence intensity peak in the H_2_O_2_-treated group (Figure 6D), indicating that PAE obviously drove ROS accumulation in *F. solani* spores. On the other hand, in the groups with spores that were treated by 1.5 mL/L PAE plus 80 mM Cys for 4 and 8 h (Figure 6E,F), there was just one fluorescence intensity peak located near 10^3^ on the horizontal axis, similar to the non-PAE treated group (Figure 6A), proving that the antioxidant Cys had eliminated the intracellular excess ROS induced by PAE. Hence, the above result demonstrated that PAE was a key driver of ROS accumulation in *F. solani* spores.

In order to analyze the role of ROS accumulation in PAE antifungal action, the survival rate of spores in every experimental group was further measured by the coating method. Figure 6H showed that PAE exhibited an ability to kill the *F. solani* spores in a concentration-dependent manner, and a treatment of 80 mM H_2_O_2_ for 8 h completely killed all the spores (*p* < 0.05). However, Cys supplementation in the PAE-treated group recovered the spore survival rate up to the level of the control group without PAE treatment. The above results strongly indicated that ROS accumulation induced by PAE plays a key role in killing *F. solani* spores.

### 3.6. Effect of PAE on Nuclear Morphometry

**Figure 7:**
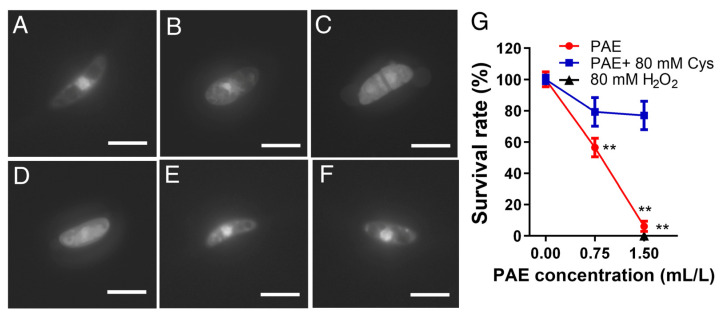
Effect of PAE on nuclear morphometry measured by DAPI staining. Spores were exposed to 0.75 (**B**) and 1.5 (**C**) mL/L PAE, 80 mM H_2_O_2_ (**D**), and to 0.75 (**E**) or 1.5 (**F**) mL/L PAE plus 80 mM Cys for 12 h. Spores without PAE treatment were served as control (**A**). Bar = 50 μm. Line chart of survival rate of spores in every experimental group (**G**). Values are mean ± standard deviations (*n* = 3). ** indicates *p* < 0.01 compared with the control group calculated by one-way ANOVA.

Nuclear morphometry was observed by DAPI staining after the spores incubated with different concentrations of PAE for 12 h. As shown in the control group (Figure 7A), the spore nuclei without PAE treatment was big and bright. However, the fluorescence intensity of the nuclei treated by 0.75 mL/L PAE became weaker and smaller than the control group (Figure 7A,B). When the PAE concentration reached 1.5 mL/L, the nuclear morphology disappeared (Figure 7C), similar to the H_2_O_2_-treated group (Figure 7D), proving that PAE drove severe nuclear damage in *F. solani* spores. Moreover, in order to estimate the relationship between nuclear damage and cell death caused by PAE, the survival rate of the spores was further quantified by the coating method. The PAE-treated concentration of 0.75 and 1.5 mL/L killed about 50 and 100% of the spores (*p* < 0.01), respectively (Figure 7G); this result was consistent with the changes of the nuclear morphology (Figure 7B,C). On the other hand, supplementation of the antioxidant Cys in the PAE-treated group was able to maintain a nuclear morphometry big and bright similar to the control group (Figure 7A,E,F), proving that the elimination of excess PAE-induced ROS contributed to keeping the nuclear architecture. Additionally, Cys supplementation had an ability to contribute to the restoration of the spore survival rate under PAE stress (Figure 7G). The above results proved that PAE treatment drove nuclear damage in the *F. solani* spores, and PAE-induced ROS accumulation acted as a critical cause of nuclear damage and spore death in *F. solani*.

## 4. Discussion

Because of having a high moisture and free sugar content, sweet potato storage roots are easily infected by *F. solani,* leading to postharvest spoilage [5,6]. However, the use of chemical fungicides such as carbendazim for the storage of this edible crop posed a potential risk to human health [32]. As a promising green preservative, the antimicrobial effects of PAE have been reported [13,33]. Although eugenol, carvacrol or cinnamaldehyde had a stronger antifungal activity than PAE in traditional contact methods [34], PAE was reported to exhibit a higher antibacterial activity against airborne microbes than these three Eos, which was tested by the vapor method using an air washer [33]. The reason why PAE exhibited a higher antimicrobial activity may depend on its high volatility. Therefore, in order to develop PAE as a novel antifungal agent used in food preservation, the antifungal activity of PAE on *F. solani* was estimated through a vapor treatment, and its preservation effect on sweet potato roots was also evaluated in the present study.

In clinical practice, *F. solani* was reported to have a high resistance to some antifungals, such as amphotericin B [24]. In order to know the antifungal activity of PAE against *F. solani*, the effect on mycelial growth was firstly evaluated in the forms of a contact and vapor treatment. In mycelium assay, the MIC against the mycelial growth of *F. solani* in the direct contact method and vapor contact method was 1.5 mL/L PAE (Figure 2A) and 0.175 mL/L air (Figure 2D), respectively. According to our previous publications, 1 mL/L of PAE could completely suppress the growth of *Aspergillus niger*, which was determined using a micro-well dilution method [35]. In this regard, PAE also exhibited effective antifungal activity against the mycelial growth of *F. solani*. Mycelia are responsible for the spoilage of fruits and vegetables. Therefore, PAE could be considered as an antifungal candidate to protect sweet potato roots from *Fusarium* root rot.

The fungus *F. solani* is widely distributed in the environment, and produces a large numbers of spores which are the major source of infectious diseases in crops and food spoilage [9]. So, the effect of PAE on the spore production of *F. solani* was further evaluated. As shown in Figure 2B and E, MICs of 1.5 mL/L PAE in the contact method and 0.175 mL/L PAE in the vapor method could also completely inhibit spore production. On the other hand, spore viability was reported to be a main cause of root colonization in plants [36]. Thus, the effect of PAE on spore viability was further tested. Additionally, PAE showed an ability to suppress the spore viability of *F. solani*. PAE concentrations reached 1.5 mL/L in the contact method and 0.15 mL/L air in the vapor method, completely inhibiting the viability of the *F. solani* spores (Figure 2C,F). Interestingly, the dose of PAE against the spore viability of *F. solani* in the vapor method was as low as one-tenth of the dose in the contact method. For instance, in the group treated with 0.1 mL/L air PAE in vapor method was 27.36% (Figure 2C), the spore survival rate was lower than 34.65% of the group treated with 1 mL/L PAE in the contact method (Figure 2F), indicating that using PAE via the vapor phase method exhibited a more efficient inhibition of spore viability than the contact method. The result was consistent with our previous research on the antifungal effect of PAE on *A. niger* (Wang et al., 2015). Hence, PAE, specially via vapor treatment, has noteworthy antifungal activity against the mycelia and spores of *F. solani*.

*F. solani* can cause not only stem lesions and end rot in sweet potatoes in the process of cultivation [37], but also, *Fusarium* root rot during the storage period [5]. *Fusarium* root rot is a kind of dry decay, and its typical lesions on sweet potato roots are circular, with light and dark brown concentric rings, causing significant economic loss during storage [5,29]. Therefore, the effect of PAE on the control of *F. solani* infection of sweet potato roots in storage was further evaluated. In this study, slices cut from the equatorial region of sweet potato roots were used to perform the pathogenicity test [3,29]. The method can ensure uniform inoculation to the central area of each sweet potato root instead of the outer layer of sweet potato flesh with varying degrees of cell development [38]. On the other hand, the slices were inoculated with mycelial plugs; such a large inoculum would magnify the extent of pathogen infection compared with the natural infection caused by fungal spores [39]. In the control group without PAE treatment (Figure 3A), the sweet potato slices seriously decayed with an occurrence of dark and sunken lesions on its surface, a symptom which is consistent with previous reports [5], whereas PAE had an ability to inhibit the decay and spoilage of sweet potato slices (Figure 3G). Therefore, the preservative assay indicated that the PAE vapor treatment contributed to controlling the *F. solani* infection of sweet potato roots.

The cell membrane is commonly regarded as an important target of EOs [40]. The cell membrane is important in maintaining cell physiology, and its main function is to protect the internal substances from not leaking to the cell exterior. The cell membrane of fungi is enriched with diverse lipids, and due to the hydrophobic character of EO constituents, the cell membrane is easily attacked by EOs resulting in an increase of its permeability [40]. PI, a kind of DNA-binding fluorescent dye, cannot enter the cell until the cell membrane structure is disrupted [41]. A flow cytometer assay showed that the spores in the control group were not stained by PI (Figure 4A); however, the spores exposed to PAE led to an obvious PI influx (Figure 4B–D), indicating that cell membrane integrity had been disrupted. The result demonstrated that PAE had the ability to damage the cell membrane function of *F. solani*, which is consistent with previous reports of *A. flavus* [21].

Mitochondria are organelle composed of two layers of plasma membrane and are reported to be another important target of EOs [34]. Mitochondria play a key role in generating cellular ATP through oxidative phosphorylation depending on the MMP, and MMP is an important indicator of changes in mitochondrial function [41]. Rh123 is a specific fluorescent probe for mitochondria in living cells, and this dye preferentially enters mitochondria based on a high value of MMP [21]. In the control group, the spores without PAE treatment were stained with Rh123, while PAE treatment led to an obvious reduction of stained spore rate (Figure 5), indicating MMP being damaged by PAE. The result indicated that PAE disrupted the mitochondrial function in *F. solani*. This similar phenomenon was reported in research on *C. fimbriata* [18].

Dysfunctional mitochondria caused by fungistats could result in the generation of excess intracellular ROS, and subsequently in cell death due to its oxidative damage to cellular macromolecules [20]. DCFH-DA is a kind of ROS-sensitive probe used for ROS detection [13]. Furthermore, the effect of PAE on the ROS level and cell viability of *F. solani* was analyzed. PAE drove ROS accumulation in cells (Figure 6B,C) and meanwhile resulted in spore death in a concentration-dependent manner (Figure 6H). It is well known that Cys is a classical antioxidant to eliminate ROS in cells. As expected, Cys supplementation restored the ROS level to a low value similar to the non-PAE treated group, and also protected cell death induced by PAE (Figure 6E–H). Therefore, the combined use of DCFH-DA staining and a spore survival test demonstrated that ROS driven by PAE plays a critical role in contributing to cell death in *F. solani*. This result was consistent with previous reports of *C. fimbriata* [18].

Usually, excessive intracellular ROS is regarded as a molecular signal to initiate apoptosis, and nuclear damage is a late marker of apoptosis [21]. In order to confirm whether nuclear damage occurred, the nuclear morphology was further observed via DAPI staining. DAPI, a DNA-specific fluorescent probe, is commonly used to detect nuclear morphology changes. A DAPI assay showed that one half of the PAE MIC caused chromatin condensation in the PAE-treated spores, and PAE MIC resulted in the disappearance of nuclear morphology in *F. solani* spores (Figure 7B,C); this result was consistent with the reduction of spore viability induced by the corresponding PAE concentration (Figure 7G). In addition, Cys supplementation could contribute to maintaining nuclear morphology, and to restoring spore viability under PAE stress (Figure 7E–G). Although previous research has proved that PAE can disrupt the nuclear morphology in *A. flavus* [21,42], this result further demonstrates that ROS driven by PAE plays a critical role in killing *F. solani* spores by causing nuclear damage.

## 5. Conclusions

In summary, this study demonstrated that PAE shows notable inhibitory effects on *F. solani* and on sweet potato decay. A PAE vapor concentration of 0.15 mL/L air markedly inhibited the mycelial growth, spore reproduction and spore viability of *F. solani*. Furthermore, a PAE vapor of 0.25 mL/L air could control the *F. solani* development in sweet potato roots during storage for 9 days at 28 °C. The possible mode of antifungal action of PAE may be dependent on its ability to induce excess ROS generation. PAE firstly disrupts the barrier property of the cell membrane leading to an increase of cell membrane permeability, and subsequently drives a decrease in MMP. Consequently, excess ROS is derived from the dysfunctional mitochondria. Finally, the excess intracellular ROS plays a critical role in triggering cell death via inducing nuclear damage characterized by chromatin condensation. This study indicates that PAE vapor possesses an effective inhibitory activity against *F. solani*. In order to develop PAE as a novel fumigant used in food preservation, more research will be needed to develop the application strategies based on its volatile features.

## Data Availability

All data sets in the current study are available from the corresponding author upon reasonable request.
